# Metallosis and Nonunion: A Case Series and Literature Review

**DOI:** 10.7759/cureus.35385

**Published:** 2023-02-23

**Authors:** Muzaffar Mushtaq, Owais A Qureshi, Anmol Dua, Sabina Khan, Meesa Mehraj

**Affiliations:** 1 Orthopedics, Hamdard Institute of Medical Sciences and Research, Delhi, IND; 2 Pathology, Hamdard Institute of Medical Sciences and Research, Delhi, IND

**Keywords:** abrasion, metal ions, pseudotumour, titanium plate, nonunion, matallosis

## Abstract

Metallosis, defined as the presence of free metal particles in the tissue, including bone and soft tissue, is a rare phenomenon seen in orthopedic practice. It is more commonly seen in arthroplasty surgeries, but its occurrence in the presence of other metal implants is also well recognized. Multiple hypotheses are suggested to explain the genesis of metallosis, but it is traditionally believed that abnormal contact between the metal surfaces leads to abrasive wear causing the release of metal particles into the surrounding tissue eliciting foreign body reactions from the body’s immune system. The consequences can be local effects, which can be asymptomatic soft tissue lesions, or lead to significant osteolysis, tissue necrosis, joint effusion, and large soft tissue masses, causing secondary pathological effects. The systemic distribution of these metal particles can also contribute to the clinical picture. The literature contains multiple case reports of metallosis following arthroplasty surgeries, but there is limited information on metallosis resulting from osteosynthesis of fractures. In this review, we are presenting our experience with a few patients who developed nonunion following the index surgeries and on revision were found to have metallosis as well. It is difficult to postulate whether metallosis was contributory to the nonunion or the other way around or whether the occurrence of nonunion in face of metallosis was a pure coincidence. Additionally, one of our patients had a positive intraoperative culture, further complicating the picture. In addition to the case series, we present a succinct review of the literature on metallosis found in previous studies.

## Introduction

Metallosis is defined as the presence of free metal particles in the tissue, including the bones and soft tissues [1]. Metallosis is a rare but well-recognized entity in orthopedics and other specialties where metal implants are placed in the body. The incidence of metallosis is not accurately known, but some studies reported incidence as high as 5.3% in all types of total hip arthroplasty (THA) [2]. In orthopedic surgery, this phenomenon is well-documented in cases of arthroplasties of major joints such as the hip, knee, shoulder, and elbow [3,4]. It is suggested that metallosis results from abnormal contact between metal surfaces, resulting in abrasive wear, leading to the presence of numerous metal particles in the tissue. This occurs first locally and subsequently, through blood and lymph circulation, at distant sites [4]. These metal particles, being foreign bodies, are engulfed by tissue macrophages/histiocytes, which subsequently release inflammatory cytokines. This, in turn, activates osteoclasts leading to local osteolysis and bone resorption [3,4]. This can lead to local effects ranging from asymptomatic soft tissue lesions to significant osteolysis, tissue necrosis, joint effusion, and large soft tissue masses (pseudotumor), which can cause secondary pathological effects [5,6]. In addition to local effects, the systemic distribution of these metal particles can cause a variety of symptoms. While there are numerous reports on metallosis following arthroplasties, there is a paucity of literature on metallosis following the osteosynthesis of fractures [6].

We are presenting a case series and literature review in this article, summarizing our current understanding of metallosis in orthopedics.

## Case presentation

Case 1

Our first patient is a 52-year-old female with no comorbidities who presented to the outpatient department (OPD) with complaints of pain and swelling in her left arm. She also reported limitations in movement in her left elbow. A year ago, she sustained trauma to her left arm in a road traffic accident. She was treated elsewhere with open reduction and internal fixation (ORIF) with plating for a fractured humerus via the posterior approach. On examination, there was a well-healed posterior scar on the posterior aspect of her left arm. There was some atrophy of the musculature of the arm, and her elbow range of motion (ROM) was between 30° and 110°. A diffuse swelling was observed in the distal third of her arm, with an increased local temperature of the skin and some tenderness on deep palpation. However, there was no active sinus or discharge.

**Figure 1:**
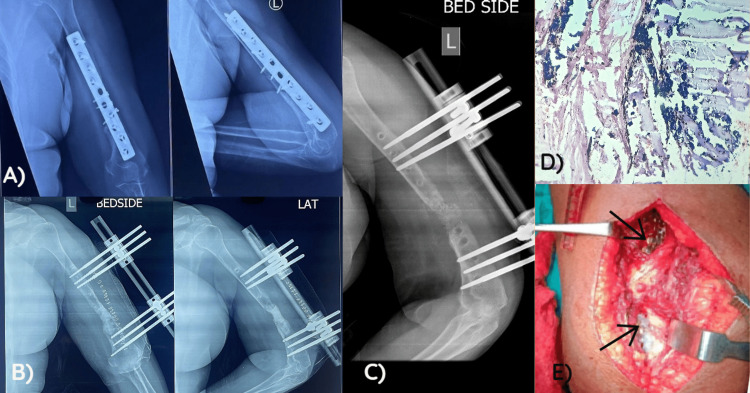
Case 1: (A) Preoperative X-ray showing nonunion with a plate in situ; (B) immediate postoperative X-ray following the first stage of the Masquelet procedure, showing debrided nonunion site filled with antibiotic cement and fixation with a rail fixator; (C) X-ray after the second stage of the Masquelet procedure, antibiotic cement removed and the gap filled with a cancellous bone graft; (D) histological examination showing metallic debris in the intracellular and extracellular spaces; and (E) intraoperative picture showing a black discoloration of the tissue around the implant (arrows pointing).

The plain radiograph of the affected arm showed a nonunion of the fracture of the distal third of the shaft of the humerus with the plate in situ. The plain radiograph showed nonunion with sclerosis of the bone ends. The blood investigations revealed a C-reactive protein (CRP) of 21.10 mg/L and an erythrocyte sedimentation rate (ESR) of 90 mm/hour, although the leukocyte count was within normal limits. She also had a Technetium-99m scan, which showed diffuse tracer uptake in the lower half of the left humerus, particularly in the lower third for about two hours. This was all suggestive of an infective nonunion, and after a detailed discussion with the patient, a staged Masquelet procedure with an external rail fixator/limb reconstruction system (LRS) was planned. In the first stage, we used the previous posterior incision and carefully dissected through a lot of scar tissue, identifying and protecting the radial nerve, down to the bone. We encountered a blackish discoloration of the tissue in close apposition to the plate. After debridement and removal of the plate, the fracture site was opened, and similarly, dark fluid of a few milliliters was seen in and around the fracture gap. Fracture ends were debrided to the healthy bleeding bone, and fixation was done with an external (rail/LRS) fixator. The intervening gap was filled with a cylinder-shaped piece of antibiotic cement containing vancomycin and added gentamicin. The tissue and fluid encountered were sent for microbiological and histopathology studies. The microscopy showed numerous polymorphs and macrophages, and the cultures grew Klebsiella. The antibiotics were changed according to the sensitivity report. The histopathology revealed infiltration with polymorphs and macrophages, many of which were laden with hemosiderin. The clinical and laboratory findings suggested a nonunion with a coexisting infection and metallosis (Figure 1).

The antibiotics were continued for six weeks, and a second procedure was planned. In the second stage, the cement was removed by carefully incising the fibrovascular membrane that had formed around the cement spacer. The defect was filled with autologous cancellous bone graft harvested from the iliac crest using the trapdoor technique to minimize donor site morbidity. Fresh samples taken at the second stage showed no growth. The patient is currently on follow-up, and serial radiographs show progressive signs of union and no active signs of infection.

Case 2

Our second patient is a 60-year-old medically fit male with a history of chronic smoking. He presented to the outpatient orthopedic department of our teaching hospital with a history of progressively increasing pain in his right upper arm. This was associated with swelling and deformity at the same site for the same period. According to the patient, he sustained trauma to his right arm about seven years ago in a road accident, resulting in a closed fracture of the proximal third of the shaft of his right humerus. This was treated with operative fixation using a plate at a different hospital. He explained that he started developing pain in the operated limb about four months ago, without any history of antecedent trauma. This pain was usually precipitated by activity, initially milder but severe enough to limit his daily activities. He also developed gradually increasing swelling in his right arm associated with visible angular deformity at the same site. The patient denied any history of fever, weight loss, or pus discharge from the surgical wound. Beyond this, there was no significant medical or surgical history.

A general physical examination revealed normal vital signs and no systemic abnormalities. A local examination of the arm showed a well-healed surgical scar on the anterolateral aspect of the right arm, approximately 20 cm long. There was significant but diffuse swelling over the proximal third of the arm, especially pronounced over the anterior and lateral aspects, with normal local temperature and no tenderness. A visible varus deformity could also be appreciated, and abnormal mobility could be demonstrated with some crepitus. The distal neurovascular exam revealed palpable pulses and no signs of nerve injury.

The patient's blood investigations were within normal limits and showed no signs of infection. Radiographs revealed a nonunited fracture of the proximal third of the humerus with a broken plate in situ and a varus and apex anterior deformity. The ends of the bone appeared sclerotic. A diagnosis of nonunion of the humeral shaft fracture with implant failure was made, and the patient was informed of this. He was thoroughly counseled about the options available and consented to open reduction and plate fixation with bone grafting.

**Figure 2:**
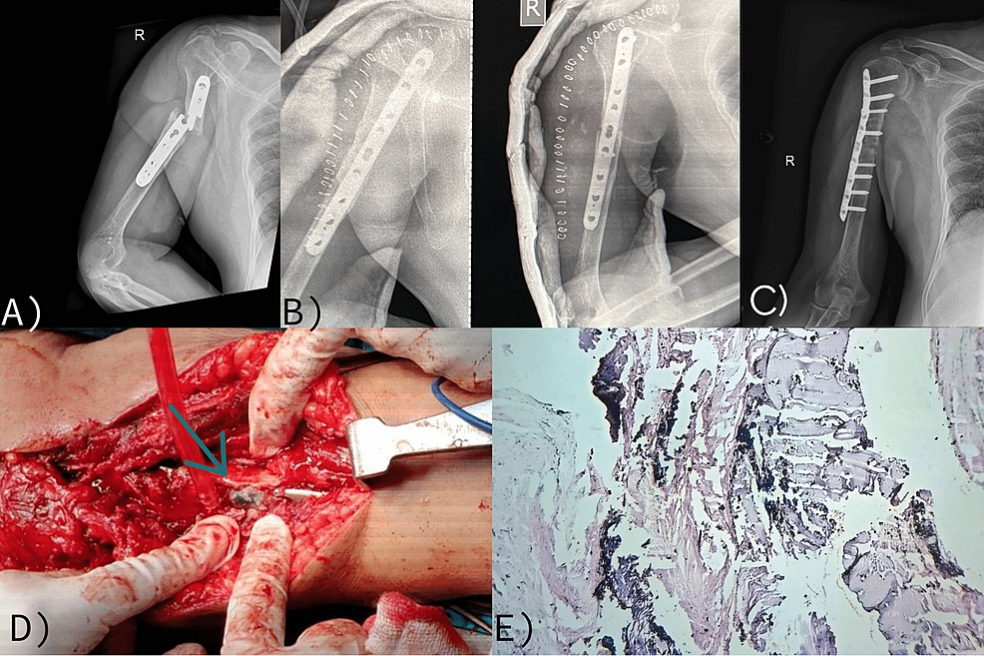
Case 2: (A) Preoperative X-ray showing nonunion of the humerus with a broken implant; (B) postoperative X-ray showing revision fixation with a plate with autologous cancellous bone grafting; (C) follow-up X-ray at about six weeks showing early graft incorporation; (D) intraoperative images showing some areas of black discoloration of tissue (arrow pointing); and (E) histological image showing features of metallosis with widely dispersed clumps of metallic particles.

He was taken to the operating room and placed under general anesthesia and positioned on a supine table with an arm board. The previous incision was used for an anterolateral approach. During the dissection, about 40 mL of dark brown, viscous fluid was drained from around the fracture site. On further dissection, a black discoloration of tissue was observed along the full length of the plate, which was debrided and sent for microbiology and histopathology. The broken implant was removed, and the fracture ends were debrided. Fixation was carried out with a longer plate, and a bone graft harvested from the iliac crest was placed around the fracture site. The wound was closed in layers. The patient's postoperative period was uneventful, and he was discharged and subsequently followed up every two weeks. Radiographs taken at the last follow-up showed positive signs of healing (Figure 2).

Microscopy showed no organisms on Gram or acid-fast bacilli (AFB) staining, and cultures were sterile. Histopathology of the sent samples revealed irregular blackish-free fragments of metallic origin, as well as discolored macrophages with focal hemorrhage, suggestive of chronic inflammation with metallosis.

Case 3

Our third patient is a 36-year-old young man who was diagnosed with osteosarcoma of the left distal femur in 2013. He was initially managed with neoadjuvant chemotherapy with cisplatin and doxorubicin and an intercalary resection of the tumor and fixed with a distal femur plate and fibular autograft and cancellous allograft in a tertiary university hospital. This was followed by three more months of chemotherapy. He stated that he was doing well for a few years after surgery when in 2017, he sustained a fall with trauma to the same lower limb. He was seen at our hospital, and radiographs showed a fracture through the same area with a broken implant. He was operated on with implant removal, freshening of the fracture, bone grafting with a fibular strut graft, and fixation with a distal femur locking compression plate (DFLCP). Again, he explained that he was able to carry out his routine activities, including work, for many years after the operation. He presented to our OPD with complaints of pain, swelling, and deformity of the left thigh for a few days after trivial trauma in the form of tripping a step. In addition to the well-healed scars of previous surgeries in the thigh and leg (for fibula harvesting for bone graft in previous surgeries), examination revealed significant swelling in the left distal thigh with varus deformity and abnormal mobility. The local temperature was normal, and there appeared to be no evidence of active infection.

**Figure 3:**
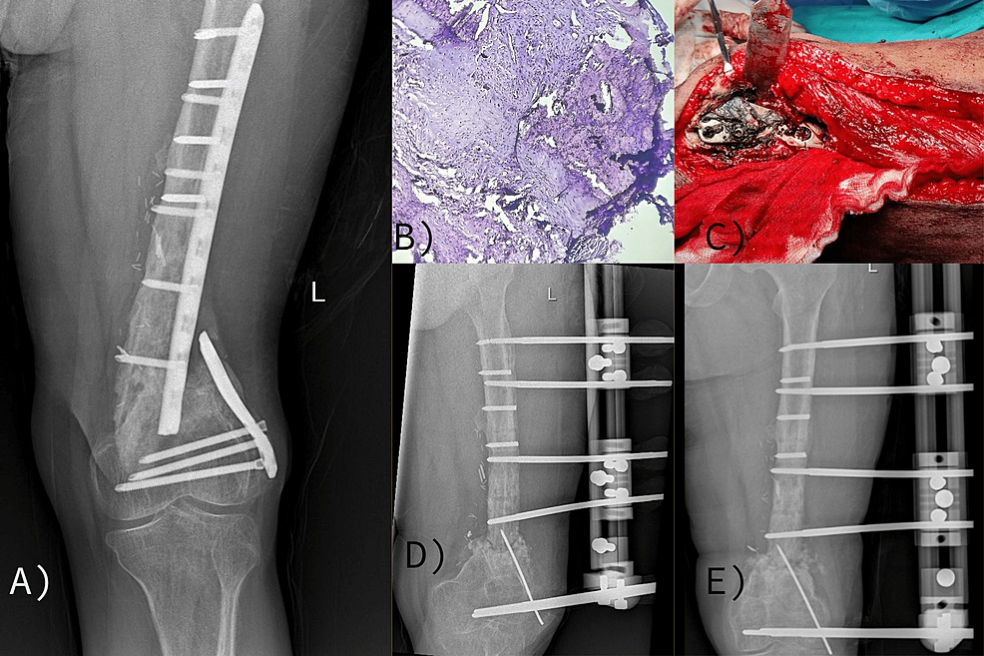
Case 3: (A) Preoperative X-ray showing nonunion at the distal femur with a broken plate in situ; (B) histological examination showing metallic particles and clumps in the tissue; (C) intraoperative picture showing a black discoloration of the tissue around the implant; (D) immediate postoperative X-ray following fixation with a rail fixator and autogenous cancellous bone grafting; and (E) follow-up X-ray at approximately 12 weeks.

Radiographs revealed a broken DFLCP with nonunion of the distal femur. Laboratory investigations showed normal leucocyte count and CRP and ESR within normal limits. After careful discussion and planning and obtaining informed consent, the patient was planned for the procedure. Using the previous scar, an incision was given. Deeper dissection revealed extensive black discoloration of soft tissue and bone surrounding the plate, and a small amount of dark brown-black-colored fluid was drained from the site. The broken implant was identified and removed, and the fracture was addressed with debridement, autologous cancellous bone grafting, and external fixation with an LRS fixator achieving good alignment in both planes. The patient is currently on follow-up and shows progressive signs of the union (Figure 3).

Case 4

Our fourth patient is a 36-year-old male with a history of chronic alcohol use. He presented to the OPD with a history of pain in the left knee and thigh for about a year and a half, which was associated with swelling and deformity at the distal thigh. In March 2019, he was involved in a road traffic accident and sustained a closed fracture of the left distal femur and a medial malleolus fracture of the same limb. He was managed in a local hospital with ORIF, using a DFLCP and cannulated cancellous screws (CCSs) for the medial malleolar fracture.

**Figure 4:**
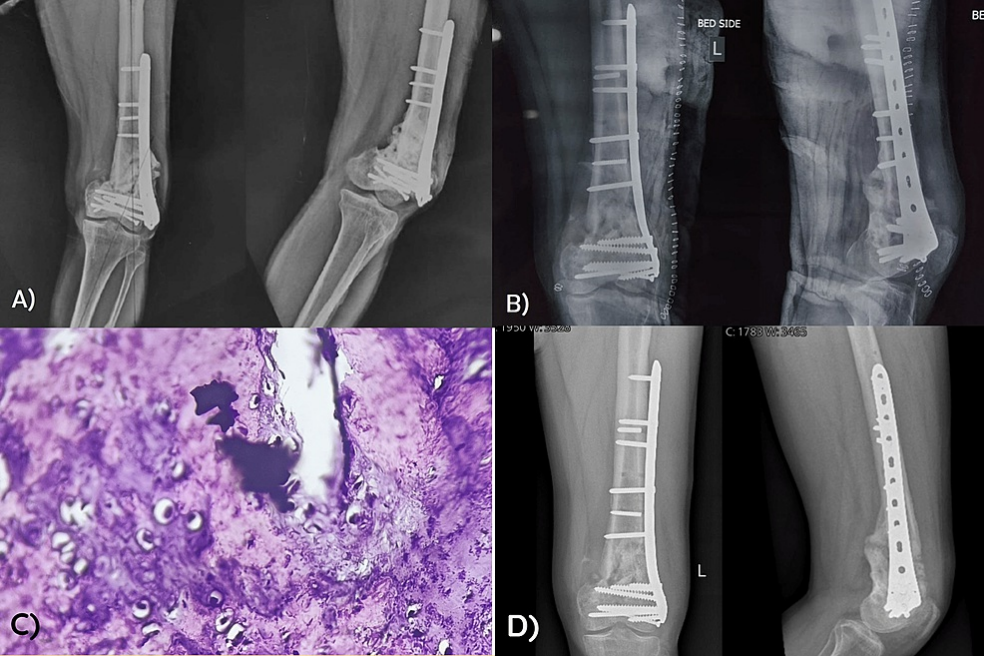
Case 4: (A) Preoperative X-ray showing nonunion in the distal femur with the implant in situ; (B) postoperative X-ray after revision fixation, correction of deformity, and autologous bone grafting; (C) histological image showing features of metallosis with intracellular metallic debris as well as large clumps; and (D) follow-up X-ray at around 12 weeks showing graft consolidation and some bridging callus.

On examination, a lateral scar was present on the anterior aspect of the distal thigh with circumferential swelling of the distal thigh. There was a visible varus alignment of the knee. The local temperature was normal, and there was no active discharge. On palpation, there was tenderness over the distal femur with palpable hardware. The ROM of the knee was 0° to 90°, and there were no motor or sensory deficits. Radiographs revealed a nonunion of the distal femur fracture with the implant in situ. Laboratory studies showed normal white blood cell counts and CRP/ESR within normal limits. After discussion and obtaining consent, the patient was planned for revision fixation with autologous cancellous bone grafting. The lateral approach was employed for the procedure. During dissection, black or deep-brown discoloration of the tissues surrounding the plate was observed. Hardware was identified and removed and revision fixation was done with a longer DFLCP after the debridement of fracture ends. An autologous cancellous bone graft harvested from the iliac crest was used. The specimens were sent for microbiological and histopathological studies. There was no growth reported from the cultural studies, but histopathology showed an extensive white blood cell infiltrate with metallic debris seen both inside and outside the tissue macrophages in the area. The wound healed well, and the fracture showed healthy signs of the union on the latest follow-up (Figure 4).

## Discussion

Metallosis is an uncommon phenomenon and, as such, poses some difficulty in diagnosis. The old medical school rule of thumb is *rare diagnoses are rarely correct*, but an orthopedic surgeon should keep this entity as a possibility in their differentials.

The clinical features in these cases are a consequence of metallosis as well as concomitant conditions such as nonunion, infection, implant failure, and neurovascular deficit. Common symptoms include swelling, occasionally large, which can mimic tumors (hence *pseudotumor*), abnormal skin pigmentation, pain, limb-length discrepancy, and painful limp or inability to use the limb. It is important to differentiate metallosis from infection, which may have similar clinical features. However, on rare occasions, they can coexist, as seen in one of our patients. Imada et al. reported a case of concomitant metallosis and prosthetic joint infection with Cutibacterium acnes in a 64-year-old patient, nine months after undergoing a THA with a dual mobility prosthesis [7]. Additionally, there are some case reports of co-occurring prosthetic joint infections with metallosis in total joint arthroplasties, with various hypotheses to account for the supposed increased susceptibility of infections in metallosis [8,9]. In addition to clinical confusion, the biochemical studies in metallosis and infections may show similar results with a raised leucocyte count and elevated ESR and CRP. A differentiating feature is a presence of raised serum ion levels in cases of cobalt-chromium (Co-Cr) prosthesis, which offers some help in the diagnosis of metallosis. Radiographic features of both are similar and nonspecific, with the presence of bone resorption and osteolysis seen in both. Some authors suggest a few typical signs pointing toward metallosis: (1) periprosthetic soft tissue amorphous densities, referred to as the *cloud sign*; (2) the *metal-line sign*, a thin rim of linear increased density in the suprapatellar pouch region in knee arthroplasty; and (3) the *bubble sign*, curvilinear radiodensities that outline the joint space due to metallic debris. However, their clinical reliability is not well-documented [10,11]. Computerized tomography (CT) and magnetic resonance imaging (MRI) may be better at defining bony and soft tissue lesions, but their use is restricted due to poor accessibility, higher costs, and sometimes due to the presence of a contraindication (incompatible metal implant for MRI).

Local responses to metal debris present in a varied manner, ranging from small, asymptomatic tissue lesions to severe destruction of bone and soft tissues. These responses are designated as metallosis, adverse reactions to metal debris (ARMD), aseptic lymphocytic vasculitis-associated lesion (ALVAL), and pseudotumors in the literature [12]. The development of metallosis with mass-like osteolysis, as well as nonunion, after ORIF, is unusual. Kang and Stern reported a case of metallosis associated with humeral hypertrophic nonunion after titanium flexible intramedullary nail insertion [13]. This patient also had radial nerve palsy, which was managed with tendon transfers. The authors, however, could not establish the chronology of events - whether nonunion was the primary event or secondary to metallosis. The authors speculated that the production of titanium alloy wear particles may have generated a net catabolic or osteolytic impact at the fracture site, contributing to the humeral non-union. However, they surmised that it is equally plausible to hypothesize that nonunion took place first, allowing the sustained motion to produce the wear debris that caused the metallosis [12]. Jones et al. [14] posited that the junctional surface area between plates and screws is small, extracortical, and away from the bone and is, therefore, unlikely to result in corrosion products in large enough quantity to cause extensive osteolysis. However, many case reports, including ours, demonstrate metallosis following ORIF with a plate, showing that it is possible to see metallosis in cases with plate-and-screw constructs. This theory may account for the fact that metallosis is much more common in arthroplasties where the metal-on-metal interfaces present huge surface areas for abrasion to occur. Mitchell et al. [15] reported metallosis of both shoulders following bilateral resurfacing hemiarthroplasty with cobalt-chromium-molybdenum alloy (Co-Cr-Mo). He was previously operated on for shoulder instability with bilateral open and arthroscopic stabilization procedures using glenoid suture anchors of titanium. During the arthroplasty, the metallic suture anchors were left in situ as they could not be accessed. The radiographs showed significant glenoid osteolysis, loosening of the implant, and near-total erosion of the anchors by the humeral prosthesis. During the subsequent revision to total shoulder arthroplasty, intraoperative findings were suggestive of metallosis, later confirmed by histopathological examination.

Preoperative aspiration and histology can help in the diagnosis, but it is not always available or possible. The diagnosis is suspected intraoperatively by the presence of metal-stained fluid or discolored soft tissue [2]. Histology can be an important diagnostic tool and will usually show necrotic and hemorrhagic tissues with a mixed inflammatory infiltrate of lymphocytes, plasma cells, and macrophages with intracytoplasmic metal debris [14,15]. Microscopic examination may also show pathogens in cases of concomitant infection, but prolonged culture and sensitivity studies will remain the gold standard in the detection of infection, which was positive in one of our cases. There is some evidence to suggest a positive correlation between metallosis and infection in total joint arthroplasty. Prieto et al. found a higher rate of infection in their metal-on-metal (MoM) THAs (DePuy ASR, Raynham, MA, USA) at Mayo Clinic, with 5.6% prosthetic joint infections compared to their baseline incidence of 1.3% [16]. While the exact cause is unclear, one hypothesis suggests that the increased local concentration of proinflammatory cytokines caused by metal debris may have a role [17]. Anwar et al. demonstrated that particulate debris of any origin acts as a scaffold to allow bacterial colonization and biofilm formation. In vitro studies have also shown cobalt and chromium debris to promote bacterial growth [17].

Park et al. [18] carried out a retrospective study on 69 patients with lateral malleolus fractures managed with ORIF with distal fibula locking plate and subsequent implant removal. Their study revealed some interesting findings regarding metallosis. Out of the total sample, they found evidence of metallosis in four of their patients, one of whom also showed osteitis. Interestingly, metallosis was seen only at the locking hole and screw in all cases, not the cortical screws, especially when there was a misalignment between the screw head and locking hole during surgery, allowing greater micromotion and subsequent corrosion. Additionally, metallosis was seen only in fractures fixed with stainless steel plates and not in those fixed with other plates made of titanium alloy or commercially pure titanium (cpTi) plates. This is possible because of higher surface corrosion and a greater number of corrosion particulate released both locally and systemically [18]. Similarly, Hayes and Richards [19] also observed that stainless steel implants show higher rates of corrosion and higher tissue absorption of metallic ions, indicative of the main components of the material like iron, Cr, nickel, and Mo, compared to those made from titanium.

## Conclusions

Metallosis is an uncommon phenomenon that may pose a diagnostic challenge. When working up a patient for infection or infected nonunion, the possibility of metallosis should be kept in mind. The importance of follow-up cannot be overemphasized, as these conditions can present many months to years after the primary procedure. Although uncommon, metallosis and infection can coexist, making the diagnosis and management even more challenging. These cases can be managed with high suspicion and due diligence during the diagnostic and therapeutic phases. On occasions when nonunion and metallosis coexist, it is hard to establish a temporal causal link between the two, and mechanisms proposed to do so are tentative hypotheses. Although prolonged, the treatment of these conditions requires a due understanding of the process and addressing the coexisting issues of nonunion or infection. In addition to local effects, metallosis can cause significant systemic effects, and the key to preventing these complications is early diagnosis.
